# Characteristics and risk factors for invasive fungal infection in hospitalized patients with acute-on-chronic hepatitis B liver failure: a retrospective cohort study from 2010 to 2023

**DOI:** 10.3389/fmicb.2024.1391814

**Published:** 2024-03-27

**Authors:** Yin-Ping Wu, Feng-Cai Li, Hang-Yu Ma, Xue-Yan Yang, Jing Zuo, Yu-Xin Tian, Li Lv, Kai Wang, Yu-Chen Fan

**Affiliations:** ^1^Department of Hepatology, Qilu Hospital of Shandong University, Jinan, China; ^2^Clinical Follow-up Center, Qilu Hospital of Shandong University, Jinan, China; ^3^Hepatology Institute of Shandong University, Jinan, China

**Keywords:** ACHBLF: acute-on-chronic hepatitis B liver failure, ACLF: acute-on-chronic liver failure, HBV: hepatitis B virus, IFI: invasive fungal infection, risk factors

## Abstract

**Background and aim:**

The global burden of invasive fungal infections (IFIs) is emerging in immunologic deficiency status from various disease. Patients with acute-on-chronic hepatitis B liver failure (ACHBLF) are prone to IFI and their conditions are commonly exacerbated by IFI. However, little is known about the characteristics and risk factors for IFI in hospitalized ACHBLF patients.

**Methods:**

A total of 243 hospitalized ACHBLF patients were retrospectively enrolled from January 2010 to July 2023. We performed restricted cubic spline analysis to determine the non-linear associations between independent variables and IFI. The risk factors for IFI were identified using logistic regression and the extreme gradient boosting (XGBoost) algorithm. The effect values of the risk factors were determined by the SHapley Additive exPlanations (SHAP) method.

**Results:**

There were 24 ACHBLF patients (9.84%) who developed IFI on average 17.5 (13.50, 23.00) days after admission. The serum creatinine level showed a non-linear association with the possibility of IFI. Multiple logistic regression revealed that length of hospitalization (OR = 1.05, 95% CI: 1.02–1.08, *P* = 0.002) and neutrophilic granulocyte percentage (OR = 1.04, 95% CI: 1.00–1.09, *P* = 0.042) were independent risk factors for IFI. The XGBoost algorithm showed that the use of antibiotics (SHAP value = 0.446), length of hospitalization (SHAP value = 0.406) and log (qHBV DNA) (SHAP value = 0.206) were the top three independent risk factors for IFI. Furthermore, interaction analysis revealed no multiplicative effects between the use of antibiotics and the use of glucocorticoids (*P* = 0.990).

**Conclusion:**

IFI is a rare complication that leads to high mortality in hospitalized ACHBLF patients, and a high neutrophilic granulocyte percentage and length of hospitalization are independent risk factors for the occurrence of IFI.

## 1 Introduction

Acute-on-chronic liver failure (ACLF) is a severe syndrome of sudden hepatic decompensation in patients with preexisting chronic liver disease (Arroyo et al., 2016; Luo et al., 2023). There are no effective therapeutic options except for liver transplantation (Belli et al., 2021). However, liver transplantation is not widely carried out due to its high cost and limited availability of donor sources (Durand et al., 2019; Perricone et al., 2023). Liver failure causes high mortality, and most patients die of severe complications such as hepatic encephalopathy and gastrointestinal bleeding (Jalan et al., 2021; Solé et al., 2021). Moreover, due to impaired immune functions in these patients, bacterial and fungal infections are common and contribute to the aggressive prognosis of the disease (Fernández et al., 2018).

Approximately 296 million people are living with hepatitis B virus (HBV) infection worldwide (World Health Organization [WHO], 2023), and the disease burden is disproportionately high in East Asia and South Africa (Hsu et al., 2023). Annually, HBV-related complications, including liver failure, cirrhosis and hepatocellular carcinoma, cause approximately 820,000 deaths worldwide, and acute-on-chronic hepatitis B liver failure (ACHBLF) accounts for 470,000 deaths in the Western Pacific region overall (World Health Organization [WHO], 2021). In patients with ACHBLF, it is generally believed that a variety of acute insults, such as HBV reactivation, induce a systemic inflammatory response (Cao et al., 2019), resulting in host immune-mediated tissue damage, cell mitochondrial dysfunction (Zhang et al., 2022), and eventually multiple organ failure (Zaccherini et al., 2021). In the early stage of ACHBLF, inflammatory cytokine storms are the dominant process (Arroyo et al., 2021), followed by immune paralysis or failure in the late stage (Fernández et al., 2018; Barros et al., 2023). Pathogen-associated molecular patterns exert inhibitory effects on monocytes and lead to reduced myeloid monocyte numbers (Inoue et al., 2018; Weichselbaum et al., 2020), and defective bactericidal and antimicrobial capacity of neutrophils (Taylor et al., 2013; Tjoa et al., 2022). Moreover, metabolic dysfunction of immune cells has been shown to be a potential contributor to the onset and progression of ACHBLF (Li et al., 2022), which facilitates bacterial and fungal infection and aggravates liver injury.

Patients with ACHBLF are susceptible to infection with bacteria and fungi, which results in rapid deterioration of liver function. Unfortunately, the diagnosis of invasive fungal infection (IFI) is often delayed due to atypical clinical features, time-consuming mycological cultures and multiple deliveries (Maschmeyer et al., 2007). With fungal infection secondary to liver failure, the mortality rate is significantly increased (Wu et al., 2012; Gustot et al., 2014). However, most antifungal drugs can induce liver injury, which limits their application in patients with ACHBLF (Zhou et al., 2022). Therefore, early identification of risk factors for fungal infection is highly important for the treatment of liver failure. It has been demonstrated that sex, age, smoking history, type 2 diabetes mellitus, chronic bronchitis, steroid use, type of antibiotic, encephalopathy, and neutropenia are risk factors for fungal infection in various diseases (Wu et al., 2012; Chen et al., 2013; Lin et al., 2013; Gao et al., 2018; Chen et al., 2021). However, there is a lack of clinical investigations focusing on the risk factors for IFI in patients with ACHBLF. Therefore, our study aimed to determine the clinical characteristics and risk factors for IFI in patients with ACHBLF.

## 2 Patients and methods

### 2.1 Study subjects

The data of 956 patients who were diagnosed with liver failure at admission to the hospital were retrospectively extracted from electronic medical records using the international classification of diseases-11 code DB99.7 from January 2010 to July 2023 in the Department of Hepatology, Qilu Hospital of Shandong University. According to the inclusion and exclusion criteria, 243 patients with ACHBLF were ultimately included, 24 of whom developed IFI. The protocol of this study was approved by the medical ethics committee of Qilu Hospital of Shandong University, and the requirement for informed consent was waived (KYLL-2023-10-035). This study was registered at Clinical Trials.gov (NCT06190002).

### 2.2 Inclusion and exclusion criteria

**FIGURE 1:**
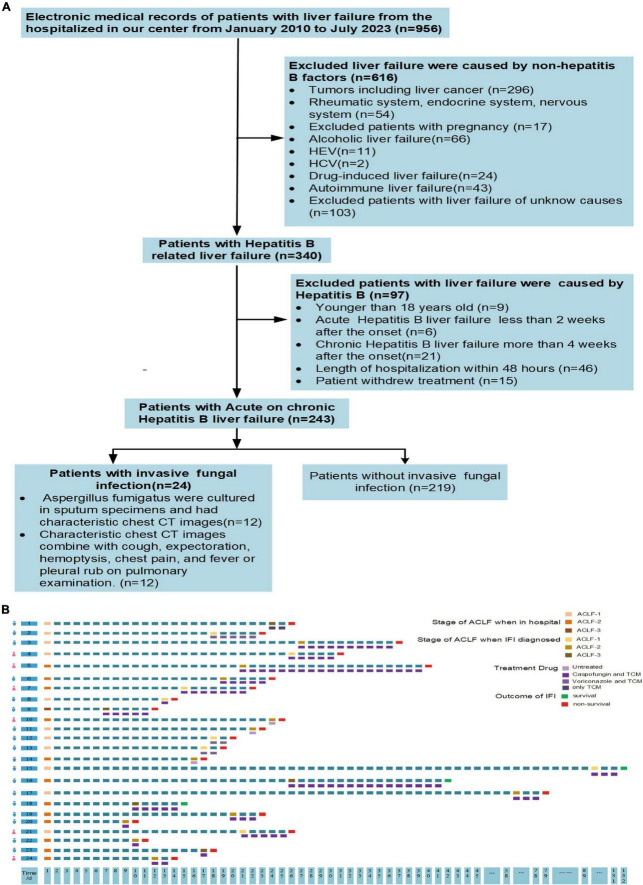
**(A)** Flow diagram of inclusion and exclusion criteria. **(B)** Characteristics of patients with invasive fungal infection. IFI, invasive fungal infection; ACLF-1, acute-on-chronic liver failure grade 1; ACLF-2, acute-on-chronic liver failure grade 2; ACLF-3, acute-on-chronic liver failure grade 3; TCM, traditional Chinese medicine.

The flowchart for the inclusion and exclusion of ACHBLF patients is shown in Figure 1A. The inclusion criteria for ACHBLF were as follows according to the Asian Pacific Association for the Study of the Liver (Sarin et al., 2019): (1) acute liver function injury [total bilirubin ≥5 mg/dl (85 μmol/L); (2) an international normalized ratio ≥1.5 or prothrombin activity ≤40%] based on previous chronic HBV infection; and (3) combined ascites and/or hepatic encephalopathy within 4 weeks after onset. The exclusion criteria were as follows: (1) died within 48 h of admission or withdrew from treatment; (2) had liver cancer combined with other extrahepatic organ malignant tumors, rheumatic diseases, or hyperthyroidism; (3) were aged <18 years; (4) had liver failure caused by alcohol, drugs, hepatitis C virus, hepatitis E virus, autoimmune liver diseases or other non-HBV viruses; (5) were pregnant; and (6) had acute liver failure or chronic liver failure.

### 2.3 Definitions of IFI

Invasive fungal infection was diagnosed according to the criteria of the European Organization for Research and Treatment of Cancer Cooperative Group on Invasive Fungal Infections and the Fungal Study Group of the National Institute of Allergy and Infectious Diseases consensus (De Pauw et al., 2008). Probable IFI was defined as follows: (1) bilateral nodular infiltrates with central cavities, halo signs, air-crescent signs, or cavities on chest computed tomography (CT) images; (2) clinical manifestations including cough, expectoration, hemoptysis, chest pain, fever or pleural rub on pulmonary examination or sputum culture positive for microbiology or patients with pulmonary CT image features that do not meet the above characteristics, but patients with a diagnosis of IFI cannot be excluded.

### 2.4 Definitions of liver failure grade

The grade of liver failure was determined according to the Asian Pacific Association for the Study of ACLF Research Consortium (AARC) (Br and Sarin, 2023). The AARC-ACLF score is composed of five main parameters, namely, total bilirubin, serum creatinine, serum lactic acid, international normalized ratio and hepatic encephalopathy, and a score ranging from 1 to 3 was assigned according to the value of each variable. Specifically, ACLF grade I (ACLF-1) was defined as 5–7 points, ACLF grade II (ACLF-2) was defined as 8–10 points, and ACLF grade III (ACLF-3) was defined as 11–15 points.

### 2.5 Data collection

The following clinical data and laboratory characteristics were collected: (1) demographic data, including age and sex; (2) laboratory data, including alanine aminotransferase, aspartate aminotransferase, total bilirubin, globulin, albumin, international normalized ratio, procalcitonin, prothrombin time activity, creatinine, platelet count, white blood cell count, neutrophilic granulocyte percentage, neutrophil granulocyte count, microculture results, and the levels of HBV markers, including HBsAg, HBeAg, and serum HBV deoxyribonucleic acid (DNA); (3) variables related to treatment drugs, including glucocorticoids and antibiotics; and (4) variables related to clinical features, including fever, hemoptysis, pleural chest pain, dyspnea, and CT image features. (5) Variables related to complications: ascites, upper gastrointestinal hemorrhage, and hepatic encephalopathy.

### 2.6 Statistical analysis

The number of patients with ACHBLF during the study period determined the smallest sample size (*n* = 115), with an estimated statistical power of 0.90 based on a two-sided <0.05 significance level when we set the incidence rate as the baseline probability to 0.02 and the alternative probability to 0.15 under a condition that assumed false-positives = 0.05. The normality of the distribution of each variable was assessed using the Anderson-Darling test. Continuous variables are presented as the means (interquartile ranges), and categorical variables are presented as numbers (percentages). The Mann-Whitney test, *t* test, chi-square test, analysis of variance, and Fisher exact test were used to determine significant differences between groups. Restricted cubic splines (RCSs) are a method used to fit and model continuous variables to create a smooth curve by splitting the range of data into many intervals and using a cubic polynomial for fitting within each interval (Lusa and Ahlin, 2020). Therefore, we used RCS analysis to model and visualize the relationships between clinical indicators and the occurrence of IFI. Univariate logistic regression analysis was performed to identify associations between each variable and the possibility of IFI, and variables with *P* < 0.2 were included in the multivariate logistic regression analysis. To further improve the prediction accuracy and the generalization ability of the algorithm, we used a machine learning method by extreme gradient boosting (XGBoost) algorithm to screen the risk factors for IFI, which is based on gradient boosting decision trees (Liu et al., 2023). In addition, SHapley Additive exPlanations (SHAP) summary plot was performed to visualize the output of the characteristics screened by XGBoost algorithm (Chen et al., 2022), and the colors in the scatter plot represent the correlation between the characteristic value and the anticipated probability (Guo et al., 2023). After screening, neutrophilic granulocyte percentage was finally identified as a main risk factor for the incidence of IFI and classified as higher (more than normal upper limit of 0.7) and normal group (less than normal upper limit of 0.7), in which the cumulative incidences of IFI in the two groups were compared by plotting Kaplan-Meier curves. *P* < 0.05 was considered to be statistical significance. All the statistical analyses were performed by EmpowerStats^1^ and R 3.4.1 software.^2^

## 3 Results

### 3.1 Characteristics of the included patients

A total of 243 patients with ACHBLF at admission were ultimately included; the median age was 48.00 (39.00, 56.00) years, and 80.25% (195/243) of these patients were men. During hospitalization, 24 ACHBLF patients (9.88%) developed IFI on average 17.5 (13.50, 23.00) days after admission. The basic characteristics of ACHBLF patients with IFI and non-IFI are presented in Table 1. Specifically, the percentages of neutrophilic granulocytes were 72.70 (63.85, 80.70) in non-IFI patients and 74.80 (71.68, 86.22) in IFI patients (*P* = 0.071). The plasma sodium level of non-IFI patients [136.00 (132.00, 139.00) ng/ml] was significantly greater than that of IFI patients [134.50 (131.00, 136.00) ng/ml, *P* = 0.024]. The procalcitonin level of non-IFI patients [0.45 (0.29, 0.82) ng/ml] was significantly lower than that of IFI patients [0.58 (0.36, 1.22) ng/ml, *P* < 0.001]. In addition, the length of hospitalization of non-IFI patients was 13.00 (9.00, 19.50) days, which was significantly less than that of IFI patients [21.50 (16.75, 28.50) days, *P* < 0.001].

**TABLE 1:** Characteristics of the included patients with acute-on-chronic hepatitis B liver failure.

Variables	Overall (*n* = 243)	Non-IFI (*n* = 219)	IFI (*n* = 24)	*P-*value
**Basic information**				
Gender (%)				0.496
Male	195 (80.25%)	177 (80.82%)	18 (75.00%)	
Female	48 (19.75%)	42 (19.18%)	6 (25.00%)	
Age, year, (mean ± standard deviation)	48.23 ± 11.71	48.00 ± 11.43	50.38 ± 14.09	0.347
HBsAg, IU/ml, (Q1, Q3)	2764.00 (565.13, 6086.00)	2512.00 (538.79, 6147.50)	3110.75 (1164.91, 5685.75)	0.786
HBeAg, s/co, (Q1, Q3)	14.59 (0.41, 198.74)	13.84 (0.42, 207.78)	22.16 (0.31, 133.60)	0.387
logqHBV DNA	4.93 (3.94, 6.16)	4.95 (3.93, 6.06)	4.78 (4.01, 6.51)	0.872
Cirrhosis (%)				0.935
No	93 (38.27%)	84 (38.36%)	9 (37.50%)	
Yes	150 (61.73%)	135 (61.64%)	15 (62.50%)	
Ascites (%)				0.401
No	73 (30.04%)	64 (29.22%)	9 (37.50%)	
Yes	170 (69.96%)	155 (70.78%)	15 (62.50%)	
Neutrophilic granulocyte percentage,%, (Q1, Q3)	72.90 (64.45, 81.30)	72.70 (63.85, 80.70)	74.80 (71.68, 86.22)	0.071
Neutrophilic granulocyte percentage category (%)				0.591
40–75	5 (2.06%)	4 (1.83%)	1 (4.17%)	
<=40	130 (53.50%)	119 (54.34%)	11 (45.83%)	
>=75	108 (44.44%)	96 (43.84%)	12 (50.00%)	
Neutrophilic granulocyte, 10^9/l, (Q1, Q3)	5.10 (3.25, 7.65)	4.97 (3.16, 7.63)	6.46 (3.74, 9.20)	0.218
Neutrophilic granulocyte category (%)				0.266
1.8–6.3	14 (5.76%)	13 (5.94%)	1 (4.17%)	
<=1.8	144 (59.26%)	133 (60.73%)	11 (45.83%)	
>=6.3	85 (34.98%)	73 (33.33%)	12 (50.00%)	
Length of hospitalization, day, (Q1, Q3)	13.00 (9.00, 20.50)	13.00 (9.00, 19.50)	21.50 (16.75, 28.50)	<0.001
Sodium, mmol/l, (Q1, Q3)	136.00 (132.00, 138.00)	136.00 (132.00, 139.00)	134.50 (131.00, 136.00)	0.024
Procalcitonin, ng/ml, (Q1, Q3)	0.46 (0.29, 0.82)	0.44 (0.29, 0.82)	0.57 (0.38, 1.20)	<0.001
**Liver function**				
Alanine aminotransferase, U/l, (Q1, Q3)	142.00 (74.00, 354.50)	145.00 (74.50, 354.50)	140.50 (73.50, 322.75)	0.510
Alanine aminotransferase category (%)				0.824
<=50	34 (13.99%)	31 (14.16%)	3 (12.50%)	
>50	209 (86.01%)	188 (85.84%)	21 (87.50%)	
Aspartate aminotransferase, U/l, (Q1, Q3)	128.00 (78.50, 237.50)	128.00 (80.50, 237.00)	121.00 (74.75, 273.00)	0.668
Aspartate aminotransferase category (%)				0.716
<=40	16 (6.58%)	14 (6.39%)	2 (8.33%)	
>40	227 (93.42%)	205 (93.61%)	22 (91.67%)	
Total bilirubin, umol/l, (Q1, Q3)	274.00 (199.00, 399.15)	271.50 (193.65, 399.15)	286.80 (235.72, 402.90)	0.245
Total bilirubin category (%)				0.114
<=181	63 (25.93%)	60 (27.40%)	3 (12.50%)	
>181	180 (74.07%)	159 (72.60%)	21 (87.50%)	
Albumin, g/l, (Q1, Q3)	33.20 (30.30, 35.80)	33.20 (30.30, 35.80)	33.40 (30.65, 35.60)	0.761
Albumin category (%)				0.218
40–55	16 (6.58%)	13 (5.94%)	3 (12.50%)	
<=40	227 (93.42%)	206 (94.06%)	21 (87.50%)	
Globulin, g/l, (Q1, Q3)	27.80 (23.45, 34.15)	28.40 (24.05, 34.35)	24.50 (20.43, 30.95)	0.075
Globulin category (%)				0.122
<=40	223 (91.77%)	199 (90.87%)	24 (100.00%)	
>40	20 (8.23%)	20 (9.13%)	0 (0.00%)	
**Kidney function**				
Creatinine, μmol/l, (Q1, Q3)	59.00 (48.00, 71.00)	59.00 (48.50, 71.00)	55.00 (41.25, 66.50)	0.823
**Coagulation function**				
Prothrombin time activity, %, (Q1, Q3)	33.00 (26.00, 43.00)	33.00 (26.00, 43.00)	33.00 (26.75, 42.50)	0.972
International normalized ratio, (Q1, Q3)	2.10 (1.70, 2.64)	2.11 (1.68, 2.66)	1.98 (1.78, 2.49)	0.821
International normalized ratio category				0.541
<=1.5	169 (69.55%)	151 (68.95%)	18 (75.00%)	
1.5–2.5	74 (30.45%)	68 (31.05%)	6 (25.00%)	
**Nervous system**				
Hepatic Encephalopathy (%)				0.611
No	182 (74.90%)	163 (74.43%)	19 (79.17%)	
Yes	61 (25.10%)	56 (25.57%)	5 (20.83%)	

HBsAg, hepatitis B surface antigen; HBeAg, hepatitis B e antigen; IFI, invasive fungal infection; HBV DNA, hepatitis B virus deoxyribonucleic acid. logqHBV DNA, logarithm of serum HBV DNA quantification.

### 3.2 Characteristics of patients with IFI

Twenty-four patients with ACHBLF (9.88%) developed IFI during the hospitalization period, 18 of whom (75%) were male. *The* genus Aspergillus was identified in sputum specimens from 50% (12/24) of patients; *Aspergillus fumigatus* was found in 9 (37.5%) patients, and *Aspergillus flavus* was found in 3 (12.5%) patients. The serum galactomannan test was positive in all patients, and the 1,3-β-D-glucan test was positive in 79.16% (19/24) of the patients. The median time for the diagnosis of IFI was 16.00 (11.00, 20.00) days in patients with *Aspergillus fumigatus*, and 17.00 (14.50, 19.00) days in patients with *Aspergillus flavus* (*P* = 0.878). The mortality rate of ACHBLF patients with IFI was 87.5% (21/24), which was significantly greater than that of ACHBLF patients without IFI (35.16%, 77/219), suggesting that fungal infection could be both a feature of liver failure progression and a complication with worsening liver failure, as shown in Table 2. The detailed characteristics of each patient are presented in Figure 1B. In ACHBLF patients with IFI, the median time from admission to the diagnosis of IFI was 25.00 (17.00–57.00) days in the survival group and 17.00 (14.00–21.00) days in the non-survival group (*P* = 0.037). The median age of the surviving patients was 37.00 (34.50–39.00) years, which was slightly younger than that of the non-surviving patients [49.00 (41.00–66.00) years, *P* = 0.054]. Compared with that in the survival group, the percentage of surviving patients who were positive for HBV DNA was 100% (20/20), which was significantly greater than that in the survival group [66.7% (2/3), *P* = 0.008].

**TABLE 2:** Characteristics of IFI patients.

Characteristics	Overall (*n* = 24)	Survival (*n* = 3)	Non-survival (*n* = 21)	*P-*value
Gender (%)				0.285
Male	18 (75.00%)	3 (100.00%)	15 (71.43%)	
Female	6 (25.00%)	0 (0.00%)	6 (28.57%)	
Age category, year (%)				0.089
<=48	13 (54.17%)	3 (100.00%)	10 (47.62%)	
>48	11 (45.83%)	0 (0.00%)	11 (52.38%)	
Age	48.0 (38.8, 61.5)	37.0 (34.5, 39.0)	49.0 (41.0, 66.0)	0.054
IFI diagnosis time days continuous, day	17.50 (13.50, 23.00)	25.00 (17.00, 57.00)	17.00 (14.00, 21.00)	0.037
Treatment time continuous, day	5.00 (2.00, 8.50)	16.00 (10.50, 29.00)	5.00 (1.75, 5.50)	0.012
Length of hospitalization continuous, day	21.50 (16.75, 28.50)	41.00 (27.50, 86.00)	21.00 (17.00, 26.00)	0.004
HBsAg category (%)				0.118
<=2700	10 (41.67%)	0 (0.00%)	10 (47.62%)	
>=2700	14 (58.33%)	3 (100.00%)	11 (52.38%)	
HBeAg status (%)				0.19
Negative	8 (33.33%)	0 (0.00%)	8 (38.10%)	
Positive	16 (66.67%)	3 (100.00%)	13 (61.90%)	
HBV DNA statue (%)				0.008
Negative	1 (4.35%)	1 (33.33%)	0 (0.00%)	
Positive	22 (95.65%)	2 (66.67%)	20 (100.00%)	
Neutrophilic granulocyte percentage category (%)				0.132
40–75	1 (4.17%)	0 (0.00%)	1 (4.76%)	
<=40	11 (45.83%)	3 (100.00%)	8 (38.10%)	
>=75	12 (50.00%)	0 (0.00%)	12 (57.14%)	
Neutrophilic granulocyte category, 10^9/l				0.026
1.8–6.3	1 (4.17%)	1 (33.33%)	0 (0.00%)	
<=1.8	11 (45.83%)	1 (33.33%)	10 (47.62%)	
>=6.3	12 (50.00%)	1 (33.33%)	11 (52.38%)	
Total bilirubin, umol/l	286.80 (235.72, 402.90)	202.20 (190.55, 235.25)	305.50 (251.40, 445.80)	0.164
Globulin, g/l	24.50 (20.43, 30.95)	30.50 (28.15, 34.30)	22.70 (19.30, 29.10)	0.188
Alanine aminotransferase category, U/l				0.091
<=50	3 (12.50%)	0 (0.00%)	3 (14.29%)	
>50	10 (41.67%)	3 (100.00%)	7 (33.33%)	
2	11 (45.83%)	0 (0.00%)	11 (52.38%)	
Procalcitonin category, ng/ml				0.335
<=0.1	3 (15.00%)	1 (33.33%)	2 (11.76%)	
>0.1	17 (85.00%)	2 (66.67%)	15 (88.24%)	
Use of antibiotics (%)				0.699
No	1 (4.17%)	0 (0.00%)	1 (4.76%)	
Yes	23 (95.83%)	3 (100.00%)	20 (95.24%)	
Use of glucocorticoids (%)				0.342
No	5 (20.83%)	0 (0.00%)	5 (23.81%)	
Yes	19 (79.17%)	3 (100.00%)	16 (76.19%)	
Creatinine, μmol/l	55.00 (41.25, 66.50)	37.00 (35.00, 46.00)	55.00 (46.00, 68.00)	0.477
Hemoptysis (%)				0.007
No	23 (95.83%)	2 (66.67%)	21 (100.00%)	
Yes	1 (4.17%)	1 (33.33%)	0 (0.00%)	
Dyspnea (%)				0.285
No	18 (75.00%)	3 (100.00%)	15 (71.43%)	
Yes	6 (25.00%)	0 (0.00%)	6 (28.57%)	
Pleuritic chest pain (%)				0.007
No	23 (95.83%)	2 (66.67%)	21 (100.00%)	
Yes	1 (4.17%)	1 (33.33%)	0 (0.00%)	

HBsAg, hepatitis B surface antigen; HBeAg, hepatitis B e antigen; IFI, invasive fungal infection; HBV DNA, hepatitis B virus deoxyribonucleic acid.

### 3.3 Non-linear analysis of the incidence of IFI

**FIGURE 2:**
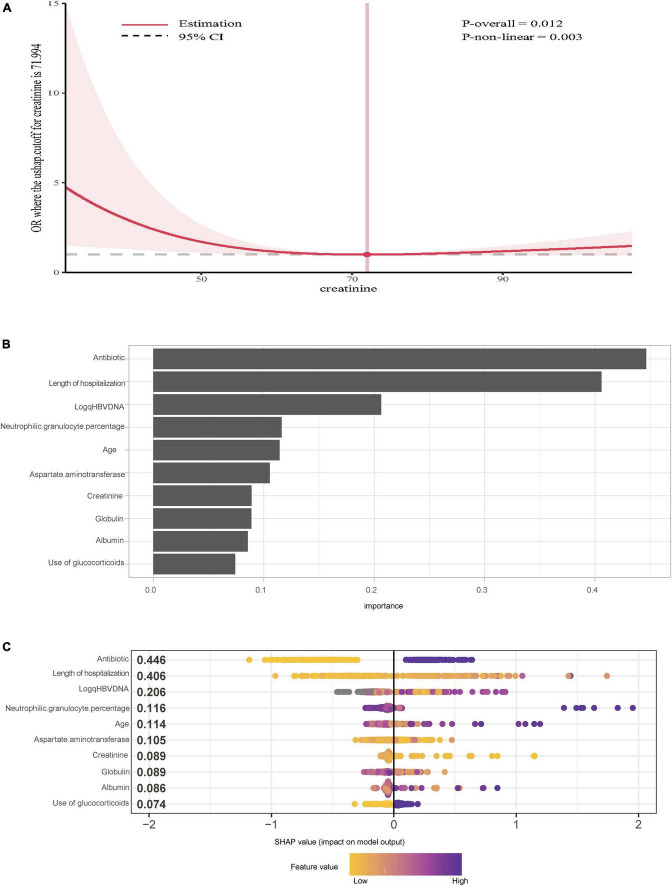
**(A)** Restricted cubic splines cure of serum creatinine and the risks of invasive fungal infection with acute-on-chronic hepatitis B liver failure. **(B)** Importance of the variables selected by extreme gradient boosting. **(C)** SHAP summary plot for the top 10 important features of the occurrence of IFI.

We performed RCS analysis to visualize the non-linear associations between potential variables and the occurrence of IFI. There was no non-linear relationship between alanine aminotransferase, aspartate aminotransferase, globulin, albumin, HBsAg, white blood cells, platelets, neutrophil granulocytes, neutrophil percentage, procalcitonin, age, total bilirubin, sodium, international normalized ratio and the occurrence of IFI (*P* > 0.05). Notably, a non-linear relationship was reported between the serum creatinine level and the risk of IFI (*P* = 0.003). As shown in Figure 2A, the OR decreased with increasing serum creatinine when the serum creatinine concentration was <72 μmol/L, and the OR increased with increasing serum creatinine when the serum creatinine concentration was >72 μmol/L.

### 3.4 Univariate and multivariate logistic regression analyses of the risk factors for IFI

As shown in Table 3, univariate logistic regression revealed that length of hospitalization (OR: 1.05, 95% CI: 1.02–1.08, *P* = 0.003), HBV DNA status (OR: 5.25, 95% CI: 0.69–40.06, *P* = 0.136), neutrophilic granulocyte percentage (OR: 1.03, 95% CI: 1.00–1.07, *P* = 0.046), and globulin (OR: 0.95, 95% CI: 0.90–1.01, *P* = 0.058) were associated with the incidence of IFI. Variables with *P* < 0.2 were selected for multivariate logistic regression, which revealed that neutrophilic granulocyte percentage (OR: 1.04, 95% CI: 1.00–1.09, *P* = 0.042) and length of hospitalization (OR: 1.05, 95% CI: 1.02–1.08, *P* = 0.002) were independently associated with the incidence of IFI (*P* < 0.05).

**TABLE 3:** Univariate and multivariate logistic regression analyses of the risk factors for IFI.

Characteristic	Univariate logistic regression		Multivariate logistic regression	
	**OR (95% CI)**	***P-*value**	**OR (95% CI)**	***P-*value**
Age, year	1.02 (0.98–1.05)	0.346	–	–
Gender (%)	1.40 (0.53–3.76)	0.498	–	–
Globulin, g/l	0.95 (0.90–1.01)	0.058	0.95 (0.90–1.01)	0.123
Albumin, g/l	1.02 (0.93–1.13)	0.652	–	–
Creatinine, μmol/l	1.00 (0.99–1.01)	0.822	–	–
International normalized ratio	1.02 (0.60–1.74)	0.953	–	–
Prothrombin time activity,%	1.02 (0.99–1.05)	0.307	–	–
White Blood Cell, 10^9/l	1.04 (0.95–1.13)	0.282	–	–
Neutrophilic granulocyte, 10^9/l	1.06 (0.97–1.16)	0.231	–	–
Neutrophilic granulocyte percentage,%	1.03 (1.00–1.07)	0.046	1.04 (1.00–1.09)	0.042
Log qHBV DNA	1.04 (0.86–1.26)	0.670	–	–
HBV DNA statue	5.25 (0.69–40.06)	0.136	4.78 (0.61–37.45)	0.137
Ascites (%)	1.12 (0.64, 1.97)	0.681	–	–
Length of hospitalization, days	1.05 (1.02, 1.08)	0.003	1.05 (1.02–1.08)	0.002

HBV DNA, hepatitis B virus deoxyribonucleic acid. The following quantitative data were obtained: age, neutrophilic granulocyte percentage, neutrophilic granulocyte count, white blood cell count, prothrombin time, globulin, creatinine, international normalized ratio, log (qHBV DNA), albumin and length of hospitalization. Categorical data: HBV DNA status, ascites, and sex.

In addition, to further verify the reliability and stability of the results, the XGBoost algorithm was used, and the 10 top variables are shown in Figure 2B. The SHAP summary plot demonstrated that the use of antibiotics (SHAP value = 0.446), length of hospitalization (SHAP value = 0.406) and log (qHBV DNA) SHAP value = 0.206 were the top three risk factors for IFI (Figure 2C).

### 3.5 Dynamic analysis of neutrophilic granulocytes, pre-albumin and globulin in patients with and without IFI

Previous studies have reported that pre-albumin (Zhang et al., 2023), globulin (Singh et al., 2019), and neutrophilic granulocytes (Wu et al., 2021) are associated with fungal infection in patients with liver failure. Based on our risk factor results for IFI, we investigated the dynamic profiles of the neutrophilic granulocyte percentage, neutrophilic granulocyte count, pre-albumin level and globulin level on Days 1, 3, 5, 8, and 12 after admission in patients with and without IFI, as shown in Table 4.

**TABLE 4:** Dynamic analysis of neutrophilic granulocyte, pre-albumin and globulin levels in patients with and without IFI.

Characteristics		Globulin, g/l	Neutrophilic granulocyte, 10^9/l	Pre-albumin, g/l	Neutrophilic granulocyte percentage,%
IFI	Day 1	28.10 (23.45, 33.85)	4.06 (3.40, 6.71)	4.50 (3.23, 5.60)	70.30 (60.85, 78.85)
	Day 3	33.80 (29.95, 35.25)*	6.86 (5.38, 8.46)	5.85 (4.28, 8.05)	82.40 (73.90, 86.40)
	Day 5	34.25 (30.7 0, 37.45)*	8.58 (6.56, 9.64)	6.60 (5.80, 8.30)	83.50 (76.73, 87.00)
	Day 8	23.30 (17.90, 28.60)	8.55 (6.71, 11.76)	7.85 (4.50, 9.65)	82.35 (77.88, 88.13)
	Day 12	23.10 (16.40, 25.85)	10.37 (8.00, 13.00)	7.40 (5.10, 11.00)	87.80 (79.90, 90.30)*
Non-IFI	Day 1	29.6 (24.90, 35.15)	4.85 (3.16, 7.23)	4.20 (3.03, 5.58)	70.60 (63.20, 79.30)
	Day 3	29.05 (24.10, 33.53)*	6.64 (4.97, 9.85)	5.30 (3.98, 7.53)	80.50 (73.70, 85.60)
	Day 5	27.15 (22.70, 31.90)*	6.80 (4.78, 10.48)	7.00 (5.00, 9.20)	80.90 (73.10, 85.50)
	Day 8	26.30 (21.90, 30.00)	7.06 (5.07, 10.67)	7.85 (5.60, 10.73)	81.20 (73.08, 86.30)
	Day 12	24.50 (19.55, 29.20)	8.60 (5.52, 12.77)	9.10 (5.83, 12.18)	81.90 (74.40, 89.30)*

*P < 0.05 (there were significant differences in globulin between the IFI group and the non-IFI group on the 3rd and 5th days after admission). The percentage of neutrophils on the 12th day after admission was significantly different between the IFI group and the non-IFI group.

**FIGURE 3:**
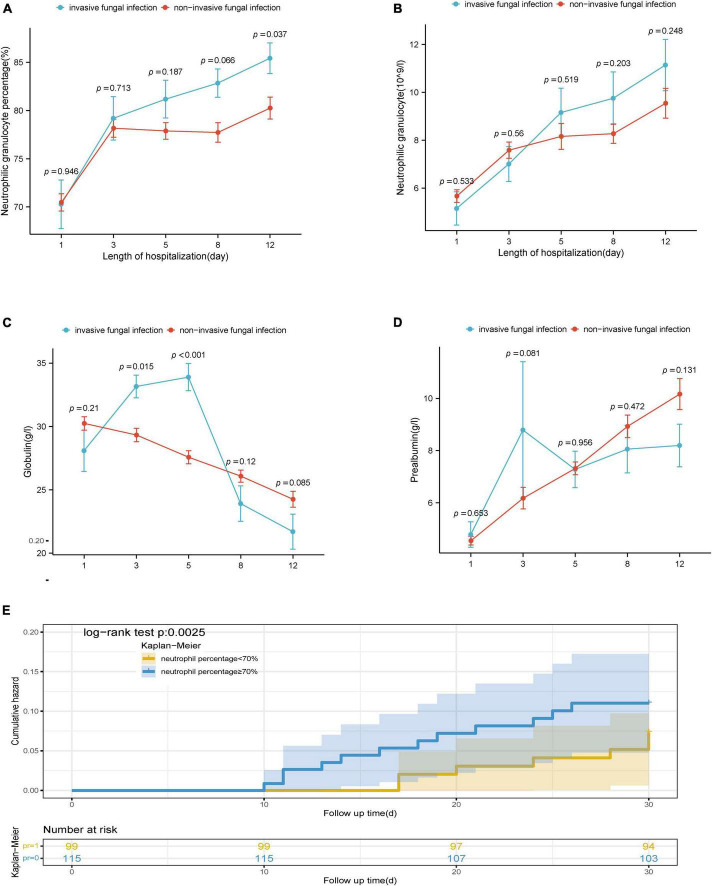
**(A)** Dynamic differences in percentage of neutrophilic granulocytes between the IFI group and the non-IFI group. **(B)** Dynamic differences in neutrophil granulocytes the between the IFI group and the non-IFI group. **(C)** Dynamic differences in globulin between the IFI group and the non-IFI group. **(D)** Dynamic differences in pre-albumin between the IFI group and non-IFI group. **(E)** Analysis of the cumulative incidence of IFI in high and low percentage of neutrophilic granulocytes groups. IFI, invasive fungal infection; non-IFI, non-invasive fungal infection; SHAP, SHapley additive exPlanations.

Specifically, the neutrophilic granulocyte percentage of patients with IFI increased from 70.30 (60.85, 78.85) on Day 1 to 87.80 (79.90, 90.30) on Day 12. The percentage of neutrophilic granulocytes in patients without IFI increased from 70.60 (63.20, 79.30) on Day 1 to 81.90 (74.40, 89.30) on Day 12. The difference in neutrophil percentage between the two groups on Day 12 was statistically significant (*P* = 0.040), as shown in Figure 3A. The number of neutrophilic granulocytes in patients with IFI increased from 4.06 (3.40, 6.71) on Day 1 to 10.37 (8.00, 13.00) on Day 12. The number of neutrophilic granulocytes in patients without IFI increased from 4.85 (3.16, 7.23) on Day 1 to 8.60 (5.52, 12.77) on Day 12, as shown in Figure 3B. Furthermore, the globulin level of patients with IFI decreased from 28.10 (23.45, 33.85) g/L on Day 1 to 23.10 (16.40, 25.85) g/L on Day 12. The globulin level of patients without IFI decreased from 29.6 (24.9, 35.15) g/L on Day 1 to 24.50 (19.55, 29.20) g/L on Day 12. The difference in globulin levels between the two groups on Day 5 was statistically significant (*P* < 0.001), as shown in Figure 3C. The pre-albumin level of patients with IFI increased from 4.50 (3.23, 5.60) g/L on Day 1 to 7.40 (5.10, 11.00) g/L on Day 12, and the pre-albumin level of patients without IFI increased from 4.20 (3.03, 5.58) g/L on Day 1 to 9.10 (5.83, 12.18) g/L on Day 12, as shown in Figure 3D. These results suggested that neutrophilic granulocyte percentage functioned as the main risk factor for the incidence of IFI. Accordingly, Kaplan-Meier analysis revealed that a higher neutrophilic granulocyte percentage more than the cut-off value of 0.7 was significantly associated with the incidence of IFI, as shown in Figure 3E.

### 3.6 Interaction analysis of glucocorticoids and antibiotics on the incidence of IFI

**FIGURE 4:**
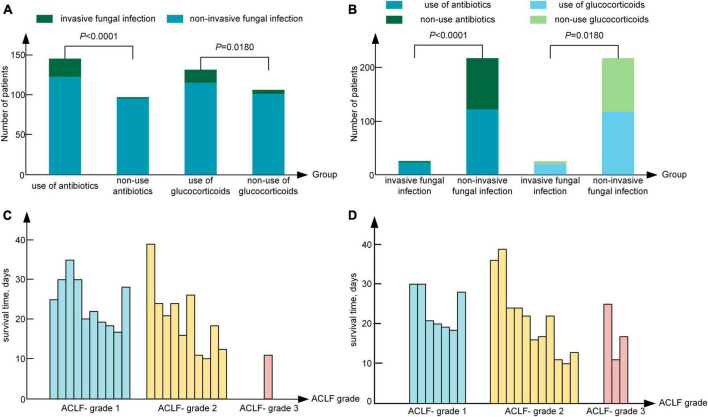
**(A)** Relationship between the use of glucocorticoids or antibiotics and the occurrence of IFI. **(B)** The use of antibiotics or glucocorticoids in the invasive fungal infection group and the non-invasive fungal infection group. **(C)** The survival time of IFI patients at admission. **(D)** The survival time of IFI patients at the time of diagnosis. IFI, invasive fungal infection; ACLF-1, acute-on-chronic liver failure grade 1; ACLF-2, acute-on-chronic liver failure grade 2; ACLF-3, acute-on-chronic liver failure grade 3.

A previous study showed that the use of glucocorticoids and antibiotics are risk factors for fungal infection in patients with ACLF (Chen et al., 2013). In our study, 56.38% (137/243) of patients had a history of glucocorticoid use, 13.87% (19/137) of whom suffered from IFI. A total of 43.62% (106/243) of patients had no history of glucocorticoid use, 4.72% (5/106) of whom suffered from IFI, which was significantly lower than that of patients who used glucocorticoids (*P* = 0.018). Moreover, 59.67% (145/243) of patients had a history of antibiotic use, 15.86% (23/145) of whom suffered from IFI. A total of 40.33% (98/243) of patients had no history of antibiotic treatment, and 1.02% (1/98) of patients suffered from IFI, which was significantly lower than that of patients who used antibiotics (Figure 4A) (*P* < 0.0001). In the IFI group, 95.83% (23/24) of patients had a history of antibiotic use, and 55.70% (122/219) of patients in the non-IFI group had a history of antibiotic use. The rate of antibiotic use of IFI group was significantly higher than that in the non-IFI group (*P* < 0.0001). The rate of glucocorticoid use was 79.17% (19/24) in the IFI group was significant higher than that (118/219, 53.88%) in the non-IFI group (*P* = 0.018), as shown in Figure 4B. Of the 24 patients with IFI, 75% (18/24) had a history of both glucocorticoid use and antibiotic use, 4.17% (1/24) had a history of antibiotic use only, and 20.83% (5/24) had a history of glucocorticoid use only. The interaction analysis revealed that the interaction effect of antibiotic use and glucocorticoid use on the incidence of IFI was not significant (*P* = 0.990).

### 3.7 Prognosis of ACHBLF patients with IFI

Among the 24 patients with IFI, 70.83% (17/24) were treated with caspofungin (50 mg ivdrip qd) and TCM, 12.50% (3/24) were treated with voriconazole (200 mg po bid) and TCM, and 4.17% (1/24) were treated with TCM only. A total of 12.50% (3/24) of patients were severely ill and did not receive treatment because of the wishes of their families. The mortality rate in the group treated with caspofungin and TCM was 82.35%, and the mortality rate in the group treated with voriconazole and TCM was 100%. However, the mortality rate of ACHBLF patients with IFI was as high as 87.5% (21/24), and the mean survival time was 21.00 (17.00, 26.00) days. Specifically, 17 patients died of respiratory failure, and 4 patients died of hepatic encephalopathy. In all the 3 patients with *Aspergillus flavus* infection, 1 patient was treated with caspofungin and TCM, 1 patient with voriconazole and TCM, and the other 1 patient withdrew treatment. In the patients with *Aspergillus fumigatus* detected by sputum culture, 8 patients were treated with caspofungin and TCM, and 1 patient withdrew treatment. The mortality rate was 77.78% (7/9) in patients with *Aspergillus fumigatus* and 100% (3/3) in patients with *Aspergillus flavus* (*P* = 0.371). As shown in Figure 4C, 47.62% (10/21) of patients were classified as ACLF grade 1, 47.62% (10/21) as ACLF grade 2, and 4.76% (1/21) as ACLF grade 3 at admission; moreover, 33.33% (7/21) of patients were classified as ACLF grade 1, 52.38% (11/21) as ACLF grade 2 and 14.29% (3/21) as ACLF grade 3 on the day of IFI diagnosis, as shown in Figure 4D. The survival time [17.00 (14.00, 21.00) days] of patients with ACLF grade 3 was significantly shorter than that of patients with ACLF grade 1 and ACLF grade 2 [21.50 (17.25, 27.50) days], and the proportion of IFI patients increased from 4.76% at admission to 14.29% at the time of IFI diagnosis in ACLF grade 3.

## 4 Discussion

The high-risk groups for IFI include patients with cancer and immunocompromised diseases who received stem cell transplantation or liver transplantation (Pacholczyk et al., 2011; Zicker et al., 2011). In recent years, patients with ACLF have been reported to be prone to fungal infection of the respiratory and urinary system due to immunological paralysis of the later stage of disease, which results in disease aggravation and poor prognosis (Wang et al., 2011; Chen et al., 2013; Gao et al., 2018). Here, we described the clinical characteristics of hospitalized ACHBLF patients with IFI and reported that pulmonary fungal infection was the predominant cause of IFI. Multiple logistic regression analysis revealed that the neutrophilic granulocyte percentage and length of hospitalization were independent risk factors for IFI. Machine learning XGBoost provides a more comprehensive set of feature importance measures, and increases the interpretability of the model and calculate the contribution value (Jiang et al., 2021). Moreover, the XGBoost algorithm provides a more automated method to avoid overfitting (Lee et al., 2023). Therefore, we used the machine learning XGBoost algorithm to screen risk factors for the occurrence of IFI, which revealed that the use of antibiotics, length of hospitalization and log (HBV DNA) were the three most important independent risk factors for IFI. Importantly, the effects of antibiotics and glucocorticoids on the incidence of IFI were evaluated via interaction analysis. Furthermore, we demonstrated that the serum creatinine level showed a non-linear association with the possibility of IFI by RCS method. The dynamic changes in pre-albumin, globulin, neutrophil percentage and neutrophil count in the IFI and non-IFI groups were compared on days 1, 3, 5, 8, and 12 after admission. In our present study, the incidence of IFI in ACHBLF patients was 9.88%, and the mortality rate was 87.5%, which is in agreement with previous reports (Wang et al., 2011; Schmiedel and Zimmerli, 2016; Verma et al., 2019). Furthermore, the mortality rate (87.5%) of ACHBLF patients with IFI was significantly greater than that (35.16%) of ACHBLF patients without IFI, suggesting that IFI aggravated the prognosis of liver failure. Therefore, IFI should be warranted in the management of ACHBLF.

Due to the real-world limited mobility rate of IFI in AHCBLF patients, the risk factors for the incidence of IFI have rarely been reported. It has been reported that activated neutrophils are involved in the formation of neutrophil extracellular traps, which are reportedly associated with poor prognosis in patients with ACLF (von Meijenfeldt et al., 2022). Another previous study showed that the ratio and number of neutrophils were sensitive biomarkers of the prognosis and outcome of liver cirrhosis and liver failure (Rice et al., 2018; Li et al., 2021), and an increased circulating neutrophil count was associated with 28-day mortality in patients with ACLF (Wu et al., 2021). However, the dynamic changes of neutrophil percentage and counts have not been reported. In our study, the neutrophil percentage of IFI patients was significantly higher than that of non-IFI patients at the 12nd day of admission, rather than the count of neutrophil. Accordingly, higher neutrophilic granulocyte percentage more than the cut-off value of 0.7 showed higher cumulative incidence of IFI compared with patients with normal neutrophilic granulocyte percentage. These results supported that neutrophilic granulocyte percentage functioned as the main risk factor for the incidence of IFI. The observations can be well explained by the fact that neutrophils are crucial in host protection against invasive candidiasis and aspergillosis (Desai and Lionakis, 2018). Understanding the molecular mechanism that mediate protective neutrophil recruitment and effector function against infungal infection is essential for immune-based strategies in ACHBLF patients.

A study suggested that compared with a hospitalization duration less than 7 days, patients hospitalized for more than 7 days are more likely to have methicillin-resistant *Staphylococcus aureus* infection (Nguyen et al., 2010). Our results showed the average period on the diagnosis of IFI was 17.5 (13.50–23.00) days after admission. Obviously, the length of hospitalization in the non-survival group was longer than that in the survival group (*P* = 0.004). These results can be explained by the fact that length of hospital stay increased the risk for the exposure to pathogenic fungi in hospital, especially for the patients in immunocompromised statue (Biondo et al., 2012). Therefore, ACHBFL patients who are long stayed in hospital should be alert to fungal infection, and the enlargement for the disinfection of hospital ward should be managed. Reactivation of HBV replication is an important cause of ACHBLF (Kim et al., 2021), and leads to the exhausted HBV-specific T cell immunity, which can increase the susceptibility to fungal infection (Ratnam and Visvanathan, 2008). Previous studies have also shown that a serum HBV DNA concentration greater than 10^3^ IU/ml is a risk factor for IFI in patients with ACHBLF (Lin et al., 2013). Using a machine learning XGBoost algorithm, we found that high HBV DNA load was also a risk factor for IFI, suggesting that antiviral therapy was beneficial to reduce the incidence of IFI in ACHBLF patients.

Acute-on-chronic hepatitis B liver failure has been reported to be associated with systemic inflammatory markers, including white blood cells, plasma C-reactive protein, proinflammatory cytokines and chemokines (Jalan and Williams, 2002; Moreau et al., 2013; Engelmann et al., 2022). A previous study reported that the use of multiple antibiotics was an independent risk factor for the occurrence of IFI (Wang et al., 2011). Moreover, nearly 40% of ACHBLF patients exhibit a similar pathophysiological process of sepsis and oxidative stress, for which glucocorticoids might be recommended for treatment (Clària et al., 2016). During the systemic inflammatory response, the disease usually progresses to susceptibility to IFI due to impaired immune cell function (Otto et al., 2011; Patil et al., 2016). In our study, 79.17% of the patients with IFI had a history of antibiotic use, and 95.83% (23/24) of the patients had a history of glucocorticoid use. To exclude the interactive effect of antibiotic and glucocorticoid, we performed an interaction analysis and reported that the effects of antibiotics and glucocorticoids on the incidence of IFI were relatively exclusive. These results suggested that in patients receiving antibiotic and glucocorticoid treatment, clinicians should be cautious of the susceptive of IFI, especially for the patients who have the signs of fever and cough, and the increased neutrophil count percentage.

Previous studies have recommended that voriconazole should be used for the treatment of IFI, including the lowest loading dose (0.2 g po q12h) and the lowest maintenance dose (0.1 g po qd) (Gao et al., 2018). However, voriconazole has been reported to have side effects containing visual disturbances, fever, rash, and hepatotoxicity (Dolton and McLachlan, 2014), which restricted the utility of voriconazole in patients with liver injury, especially for patients with liver failure. Caspofungin is a safe rescue drug for patients with severe liver dysfunction (De Pauw et al., 2008), which has slight side effects on liver function compared with voriconazole (Wagner et al., 2006). In addition, TCM has been widely used in China and have been reported to contain potent antifungal potency and benefit for IFI patients (Dong et al., 2023; Xie et al., 2024). Therefore, most of the patients in our present study were treated by caspofungin and TCM. As the results, the mortality rate of IFI patients treated with caspofungin and TCM was 82.35%, which was lower than the 100% mortality rate in the patients treated with voriconazole and TCM. Furthermore, there were no differences of successful treatment in patients with *Aspergillus fumigatus* and *Aspergillus flavus*. Unfortunately, there is still a great lack of strong and effective treatments for IFI in patients with liver failure. The exact therapeutic effect and new drugs for treating liver failure caused by IFI are emerging.

There are several strengths of our study. First, we revealed the dynamic characteristics of invasive fungal infection in patients with ACHBLF in a large hospital-based cohort which is valuable to guide clinical practice. Second, we identified the conditions of patients who have unique risk to develop IFI in ACHBLF. However, there are also some limitations in our manuscript. First, we revealed that the most common type of fungal infection was *Aspergillus*, rather than *Candida* infection which has been previously reported in patients with liver failure (Bassetti et al., 2017). Similar with most of reported literatures, most of our patients were diagnosed according to the culture results (Chen et al., 2013; Gao et al., 2018). However, the culture usually has a certain false-negative rate, and limits the golden standard for the diagnosis of IFI. Meanwhile, CT images and the clinical presentation were also used for the diagnosis of IFI in our study, since histological biopsy was not possible in ACHBLF patients with coagulopathy (Miceli and Kauffman, 2017). Therefore, reliable methods to detect fungal species is an urgent need in clinical condition. Second, the sample size of IFI remains small due to the rare mobility of IFI in ACHBLF patients ranging from 2 to 15% (Chen et al., 2021). In the study reported by Chen et al. (2013), 39 patients developed IFI from 787 patients with ACLF, 37 of whom died even with antifungal agents, with the reported mobility and mortality similar with our results. However, we believed that the future research should focus on great sample size with multiple centers.

In conclusion, IFI is a rare complication but leads to a high mortality in hospitalized ACHBLF patients, in which a higher neutrophilic granulocyte percentage and length of hospitalization are independent risk factors for the occurrence of IFI.

## Data availability statement

The raw data supporting the conclusions of this article will be made available by the authors, without undue reservation.

## Ethics statement

The studies involving humans were approved by the Medical Ethics Committee of Qilu Hospital of Shandong University. The studies were conducted in accordance with the local legislation and institutional requirements. The Ethics Committee/Institutional review board waived the requirement of written informed consent for participation from the participants or the participants’ legal guardians/next of kin because the subject cannot be found by using human data with identifiable information to conduct research, and the research project does not involve personal privacy and commercial interests.

## Author contributions

Y-PW: Data curation, Formal analysis, Methodology, Software, Writing – original draft. F-CL: Data curation, Formal analysis, Writing – original draft, Investigation. H-YM: Data curation, Formal analysis, Writing – review and editing. X-YY: Data curation, Formal analysis, Writing – review and editing. JZ: Data curation, Formal analysis, Writing – review and editing. Y-XT: Data curation, Formal analysis, Writing – review and editing. LL: Data curation, Formal analysis, Writing – review and editing, Investigation, Resources, Software. KW: Writing – review and editing, Supervision. Y-CF: Supervision, Writing – review and editing, Conceptualization, Funding acquisition, Project administration, Resources.

## Footnotes

1
https://www.empowerstats.net/en/

2
https://www.r-project.org/
